# Temporal shifts in insect cuticular hydrocarbons in response to experimental desiccation

**DOI:** 10.1371/journal.pone.0359617

**Published:** 2026-10-13

**Authors:** Abhishek Nair, Ramakrishnan Vasudeva, Christopher Mitchell, John Hunt, David J. Hosken, Manmohan D. Sharma, Subhash Rajpurohit

**Affiliations:** 1 Division of Biological and Life Sciences, School of Arts and Sciences, Ahmedabad University, Navaranpura, Ahmedabad, India; 2 Centre for Ecology and Conservation, University of Exeter, Penryn, Cornwall, United Kingdom; 3 School of Science, Faculty of Engineering, Computing and Science, Western Sydney University, Hawkesbury Campus, Richmond, New South Wales, Australia; University of Southampton, UNITED KINGDOM OF GREAT BRITAIN AND NORTHERN IRELAND

## Abstract

Insects are vulnerable to desiccation as they are small and have a high surface area-to-volume ratios. Over 80% of their body water is lost through the cuticle, but insects possess specialised cells that synthesize cuticular hydrocarbons (CHCs) to form a hydrophobic waxy barrier that reduces water loss. However, how rapidly CHCs are synthesized and transported across the cuticle under desiccating conditions remains poorly understood. To examine this, we exposed male *Drosophila melanogaster* to varying desiccation durations (2, 4, 6, 8, and 10 h) and compared CHC profiles for each of these time-points to an experimental control (no desiccation: 0 h). We found a rapid, time-dependent shift in CHC composition, marked by an early increase in short-chain hydrocarbons followed by enrichment of longer chain, methyl-branched CHCs over longer periods of desiccation exposure. Cumulative water loss increased most sharply within the first 2 h of desiccation and then progressed more slowly from 2 to 8 h before rising again at 10 h. These changes were closely mirrored by temporal shifts in CHC composition, suggesting that CHCs are dynamic and respond rapidly to desiccation stress.

## Introduction

Terrestrial animals need to cope with a range of environmental challenges that fluctuate over space and time [1–5]. Of these, desiccation is particularly challenging. Water is globally common [6], but it may be scarce locally, and availability may vary considerably with environmental fluctuations [7]. Evaporative water loss occurs due to the vapor pressure gradient between the moist internal body environment and the drier external air, and this drives passive diffusion of water molecules outward. Water-loss challenges generate a range of adaptive strategies to protect water balance [8–11]. For terrestrial insects, their small size results in large surface-to-volume ratios, which greatly increases the risk of internal water loss.

Insects can lose water through various routes including the spiracles, orally, via excretion (urine and faeces) and through the cuticle [12], with cuticular water loss typically accounting for >80% of water loss in terrestrial insects [13–15]. For example, in queen harvester ant, changes in cuticular transpiration accounted for approximately 97% of the increase in water loss associated with nest excavation and mating [16]. The cuticular hydrocarbons (CHCs) are a complex mixture of long-chain hydrocarbons (mainly alkanes, alkenes, branched alkanes) that have primarily evolved to limit water loss via the cuticle [17]. Studies on Drosophilidae clearly demonstrate that CHC profiles vary along environmental gradients (latitudinal and altitudinal) as well as across seasons [18–20]. In general, insects with a higher proportion of longer-chain CHCs exhibit better cuticular waterproofing and consequently enhance survival under desiccation stress [21]. For instance, *Drosophila mojavensis*, a desert fly with CHC profiles rich in long-chain hydrocarbons, exhibits higher desiccation tolerance than species inhabiting less xeric environments (e.g., *D. pseudoobscura*). Similarly, the harvester ant *Pogonomyrmex barbatus,* which spends extended periods of time outside its nest and needs to withstand warm temperatures and low humidity, has a higher proportion of n-alkanes [22]. In tiger beetles *(Neocincindela perhispida campbelli)*, a species inhabiting dry environments, a greater abundance of long-chain hydrocarbons helps to reduce passive water loss from the cuticle (transpiration) [23,24]. In the aphid (*Sitobion avenae)*, deposition of long-chain monomethyl alkanes provides a better barrier to water transpiration [25], and likewise, more long-chain hydrocarbons in scorpions (*Buthus occitanus*) and tenebrionid beetles (*Eleodes armata*) have been associated with a more impermeable integument [26,27]. Such specific CHC components can offer continuous protection against dry environments, but CHC profiles may also be altered in response to environmental fluctuations [18,28].

Recent work has identified one example of adaptive CHC response where short-term non-lethal desiccation exposure led to a shift in CHC profiles in *Drosophila melanogaster* populations collected from the Western Himalayas across an elevational gradient (760–2,592 meters above sea level) [20]. Evidence from other studies also suggests that changes in CHC profiles could be responsible for reduced cuticular water loss [21,29,30]. Additionally, rapid hardening in response to desiccation is well documented in *Drosophila* species, especially in females, where short-term, non-lethal desiccation exposure results in higher desiccation tolerance [21]. Studies along latitudinal gradients have also revealed significant divergence in CHC profiles among *Drosophila* species. For instance, *D. melanogaster* populations sampled along the east coast of Australia showed greater abundance of short-chain CHCs with increasing latitude [19]. A similar pattern has been reported for *D. melanogaster* populations from the east coast of the USA [18]. In contrast, an altitudinal cline showed that flies originating from higher elevations had greater abundance of long-chain hydrocarbons [20]. Although studies have linked desiccation resistance, water balance, and CHC profiles in *Drosophila* [30–32], the temporal changes in CHCs in response to desiccation are less well established.

To address this gap, we examine the CHC profiles of male *D*. *melanogaster* when exposed to different durations (2, 4, 6, 8, and 10 h) of experimental desiccation. We restricted this study to males because they are smaller than females and are more sensitive to desiccation due to their larger surface area-to-volume ratio [33]. Additionally, post-mating, females undergo a cascade of metabolic changes, possibly impacting CHC synthesis [17]. Finally, with a few exceptions, the majority of CHCs are the same in males and females [20]. Here, we hypothesize that cumulative water loss would be greatest during the initial phase of desiccation – before animals have the chance to adjust CHC profiles – with subsequent slowing as CHC composition shifts dynamically toward longer-chain length CHCs that provide increased waterproofing.

## Materials and Methods

### Fly collection and maintenance

Wild *D. melanogaster* adults were collected from orchards on agricultural land at the collection coordinates described below, with permission from respective landowners. The collections were undertaken for non-commercial academic research, did not involve any protected areas, and required no government permits or specific collection approvals under the applicable regulations in India; *D. melanogaster* is not listed as a protected species under India’s wildlife protection framework. As *D. melanogaster* is an invertebrate species and the study did not involve vertebrate animals, no animal ethics approval was required by the host institution. The flies were subsequently maintained under laboratory conditions at Ahmedabad University. A single, large population of *D. melanogaster* was founded from wild-caught flies from the Western Himalayas (Rohru, Himachal Pradesh, India; 31°N, 77.7°E, altitude 2592 meters above sea level) [20]. Briefly, wild-caught gravid females (N = 37) were individually housed in narrow *Drosophila* culture vials (30 ml) containing standard corn-meal medium [34] for 2 weeks and maintained at a constant temperature of 23^o^C under a 12 h:12 h light: dark cycle to establish a total of 37 isofemale lines via full-sib mating. To generate genetically diverse population, 10 males and 10 females from each isofemale line were pooled into a large Plexi-glass cage (37 IF lines x 20 flies = 740 adults: 370 males and 370 females). The resulting population was maintained at a size of ca. 1000–1500 individuals with non-overlapping generations. Adults were allowed to freely interact and mate for five generations before being used in this study. The population cage was housed at an ambient temperature of 23°C, following the standard rearing conditions described above. All the flies used in this study were from generation six in order to minimize any carry-over maternal effects that could impact findings [35,36].

### Experimental design

For egg collection two food plates containing corn meal agar medium were placed in the 6th generation population cage for 3 h. Following this, eggs in batches of 30 were transferred to 30 separate vials, and all the vials were placed in an incubator set at 23^o^C, 60% relative humidity and under constant light exposure. Upon emergence, 600 virgin males were collected and aged for 3 days in groups of 10 (i.e., 10 males per vial; 60 vials in total). These sixty vials were designated as follows: one experimental control group (0 h) and five desiccation treatment durations (2, 4, 6, 8, 10 h), with 10 replicate vials per group (Fig 1).

**Fig 1:**
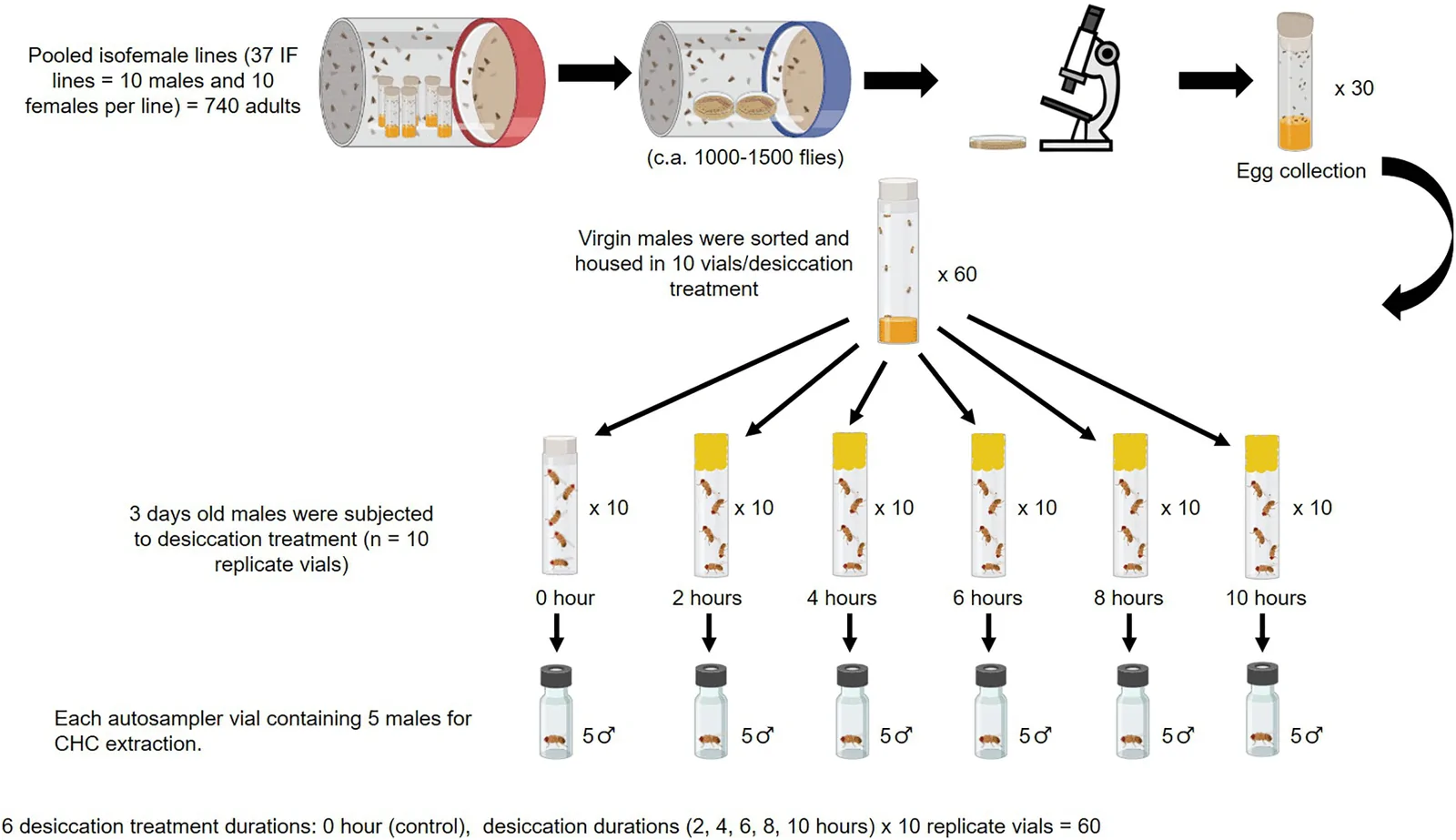
The experimental design. A population of *D. melanogaster* was established by pooling 37 isofemale lines. From this, virgin males were collected and housed in groups of 10 per vial for 3 days. Three-day old flies were then exposed to desiccation (RH < 2%) for different durations (2, 4, 6, 8 and 10 h) with 10 independent replicate vials per treatment, including a control (0 h) where flies were not exposed to dry air (total number of flies = 600). Five males from each replicate vial were pooled for CHC extractions.

### Desiccation assay

Three-day old, virgin male flies were sorted under a binocular stereo-zoom microscope (S9i, Leica Microsystems, Germany) using a mild stream of CO_2_ anaesthesia a day prior to the assay. The experimental setup included one control group (no desiccation exposure = 0 h) and five desiccation treatment durations, each with independent replicate vials (N = 60 vials total; n = 600 flies). Prior to the experiment all flies were kept in fresh food for 24 h. The control vials were not exposed to dry air (0 hours), whereas the desiccation treatment groups were exposed to dry air. To keep the air dry in the vials, 2 g of silica gel (Sigma Aldrich, USA) was added to each desiccation treatment vial, and all the vials were sealed with parafilm (Parafilm M, USA).

### Sampling across desiccation durations

Flies from the experimental control group were immediately transferred to 1.5 ml micro-centrifuge tubes, snap-frozen in liquid nitrogen, and stored at -20^°^C. Similarly, flies from the desiccation treatment groups were snap-frozen at the treatment-appropriate time points (2, 4, 6, 8, and 10 h) post-desiccation exposure and stored at -20^o^C until CHC extractions were performed. The longest desiccation durations were approximately at the point where male deaths start to occur in significant numbers (LT_50_), indicating that they are stressful [37].

### Determination of body mass and cumulative water loss

Wet body mass (mg) of adult flies stored in 1.5 ml micro-centrifuge tubes was measured by taking them out of the freezer (maintained at -20^°^C). Ten independent groups of flies (N = 5 in single sex group) from each time point were transferred onto a small aluminium weighing boat (2 x 2 cm) and measured using a OHAUS microbalance (OHAUS Corporation, USA: PAG413C) with a precision of 0.001 g. For dry mass measurements, the same flies were dried overnight in an oven at 55^°^C (± 0.5^°^C). Total body water content (*m*) was obtained as the difference between wet mass (*w*) and dry mass (*d*) (i.e., *m* = *w* – *d*). Water loss was quantified using Wharton’s method [38]. The proportion of water remaining at each desiccation time-point was calculated as *m*_*t*_ */m*_*0*_ where *m*_*t*_ is the water content at time point *t* and *m*_*0*_ is mean water content of the control (0 h) group. Cumulative water loss (%) was then calculated as [$\text{1}-(\frac{mt}{m\text{0}})$] x 100 and compared among desiccation durations.

### Chemical analyses of male CHC extracts

CHC extracts were obtained from −20^°^C stored pooled samples (5 males’/replicate vial). Flies were transferred from 1.5 ml Eppendorf tubes to autosampler vials (9 mm screw vial, 12 x 32 mm) consisting of a glass insert (0.1 ml insert, 29 x 5.7 mm) containing 100 μl of hexane. After 4 minutes (min) of soaking, vials were vortexed for 1 min to maximize extraction. Following this, flies were removed from the vials using a fine sterilized needle. The vials were dried under a nitrogen stream and stored at −20^°^C prior to CHC analysis. CHC extraction from all the samples was done in a single day.

To determine the CHC profiles of males, we used Gas Chromatography-Mass Spectrometry (GC-MS). Pentadecane was used as an internal standard because its retention time did not overlap with any of the other known hydrocarbon components of *D. melanogaster*. A one microliter hydrocarbon extract was injected into a gas chromatograph- mass spectrometer (GC-MS) (Agilent 7890A GC coupled with an Agilent 5975B MS) fitted with a DB-1 column of 30 m × 0.25 mm internal diameter × 0.25 μm film thickness, operating in a spit-less mode. Helium was used as the carrier gas at a flow rate of 1 ml/min. The inlet temperature was set at 300 °C, operating in pulsed split-less mode with a pressure pulse of 50 psi for 1 min. The initial oven temperature was held at 70^°^C for 1 min, increasing at 20^°^C/min to 180^°^C, then 4^°^C/min to 220^°^C, and 15^°^C /min to 320^°^C with a 7 min hold. The MS transfer line was set to 280^°^C. Mass spectrometry data were collected in a scan range of 50–500 *m/z*. Peak integration of hydrocarbon data was carried out using the Mass Hunter software (Agilent, version B.07.00), using ion 57 as the target ion for saturated hydrocarbons and ion 55 for unsaturated hydrocarbons. Methyl branched alkanes were identified by their mass spectra [39], and the CHC peak identities were confirmed by their retention indices [40].

To analyse differences in male *D*. *melanogaster* CHC profiles among desiccation durations, we used a multivariate approach. The relative CHC abundance of each peak was calculated by dividing its abundance by that of the internal standard (ISTD). Resulting CHC values were log_10_ transformed to ensure normality across the dataset. Multivariate outliers were assessed using Mahalanobis distances, and no significant outliers were identified [41,42]. A multivariate analysis of variance (MANOVA: of all 25 CHC peaks together) was performed with log_10_ transformed CHC relative abundance values (CHC_1_-CHC_25_) as the response variable and desiccation duration as the fixed effect in the model using *manova ()* function implemented in base R [43]. Multivariate significance was assessed using Wilks’ λ. To determine the directionality of CHC response across desiccation durations, post-hoc univariate ANOVAs were performed for individual CHC peaks, with pairwise comparisons undertaken using Tukey’s HSD test (α = 0.05). Additionally, linear regression based temporal trend analysis was performed with desiccation duration as a continuous predictor to distinguish CHCs that increased or decreased over prolonged desiccation. All statistical analyses were performed in RStudio v2025.5.0.496 [44] running R v4.5.1 [43] unless stated otherwise. The raw data underlying these analyses are provided as Supporting Information (S1 Raw data), together with an accompanying README file (S1 README).

To further visualise the results of the MANOVA and identify CHCs that strongly vary between control and desiccation treatments, we performed a variable importance analysis using a Random Forest model implemented in the *‘randomForest’* package [45]. To determine which CHCs contributed most strongly at each desiccation durations, the dataset was first split into separate subsets based on desiccation duration and each treatment duration was compared to 0 h (control). The model was built using both ‘Out of Bag (OOB) error’ estimates and training/test sets (80:20 split). We kept the 0 h (control) as constant in all the comparisons. The importance of each CHC variable was evaluated using the Mean Decrease Gini (MDG) index (importance), where higher values indicate a greater contribution of a CHC in classifying control versus desiccation treatments. The ranked importance values were visualised using bar plots, with CHC variables ordered based on their MDG scores. Cumulative water loss (%) was analysed using ANOVA, with cumulative water loss (%) as the dependent variable, and desiccation duration as the fixed effect. Tukey’s HSD test was used to compare group differences (significance; *P* < 0.05), with Boxplots showing the interquartile range (IQR), with the horizontal line denoting the sample median, whiskers describing 1.5 IQR, and raw data points shown as jittered points.

## Results

### Analysis of male CHC profile

GC-MS analysis of male *D*. *melanogaster* revealed 25 different CHCs. The profiles consisted of a combination of straight-chained alkanes, methyl-branched alkanes, and alkenes ranging in length from C_22_ to C_29_ (Table 1).

**Table 1:** Twenty-five cuticular hydrocarbons peaks were identified in male *D*. *melanogaster* with their retention indices (RI), and molecular weight (MW, Daltons). The relative abundance and variation of individual CHCs are represented as (Mean ± S.E). Compounds with different retention indices are marked with additional letters in the brackets (a, b, c, d, e, f, g, h).

CHCs	Structural formula	Retention indices	Molecular Weight	Mean	± S.E
Pentadecane ISTD	C_15_	1498	212	29133	
Docosene	C_22:1_	2187	308	0.0039	0.0002
Docosane	C_22_	2197	310	0.0439	0.0015
Branched alkane *(a)*	MeC_22_ *(a)*	2265	324	0.0048	0.0010
Tricosene *(a)*	C_23:1_ *(a)*	2271	322	0.0375	0.0013
Tricosene *(b)*	C_23:1_ *(b)*	2278	322	0.4778	0.0148
Tricosene *(c)*	C_23:1_ *(c)*	2287	322	0.0305	0.0011
Tricosane	C_23_	2298	324	0.5262	0.0124
Tetracosene *(a*)	C_24:1_ *(a)*	2374	336	0.0095	0.0004
Tetracosene *(b)*	C_24:1_ *(b)*	2377	336	0.0057	0.0002
Tetracosene *(c)*	C_24:1_ *(c)*	2382	336	0.0033	0.0001
Tetracosane	C_24_	2398	338	0.0197	0.0006
Branched alkane *(b)*	MeC_24_ *(b)*	2455	352	0.0272	0.0008
Pentacosene *(a)*	C_25:1_ *(a)*	2464	350	0.0510	0.0017
Pentacosene *(b)*	C_25:1_ *(b)*	2471	350	0.2092	0.0071
Pentacosene *(c)*	C_25:1_ *(c)*	2477	350	0.0054	0.0002
Pentacosane	C_25_	2499	352	0.1158	0.0033
Branched alkane *(c)*	MeC_25_ *(c)*	2563	366	0.0035	0.0001
Hexacosane	C_26_	2598	366	0.0029	0.0001
Branched alkane *(d)*	MeC_26_ *(d)*	2659	380	0.2385	0.0075
Heptacosane	C_27_	2699	380	0.0652	0.0020
Branched alkane *(e)*	MeC_27_ *(e)*	2747	394	0.0036	0.0001
Branched alkane (f)	MeC_28_ *(f)*	2863	408	0.2176	0.0048
Branched alkane (g)	MeC_28_ *(g)*	2874	408	0.0055	0.0003
Nonacosane	C_29_	2899	408	0.0173	0.0008
Branched alkane (h)	MeC_29_ *(h)*	2948	422	0.0279	0.0011

### Effect of desiccation duration on CHCs

The multivariate analysis revealed a strong effect of desiccation duration on CHC composition. CHC profiles differed significantly across time-points (Table 2). Follow-up univariate ANOVAs showed several individual CHC peaks (Docosane, Tricosene *(a)*, Tetracosene *(b),* Branched alkane *(c),* Branched alkane *(d),* Branched alkane *(e),* Branched alkane *(g)*) that contributed strongly to temporal differences (Table 2). Tukey’s post-hoc test revealed that these differences were primarily driven by disparities between shorter (0–2 h) and longer (6–10 h) desiccation durations (S2 Table). We performed linear trend analysis to determine whether these CHCs increased or decreased with desiccation progression. The analysis revealed that Branched alkane *(c)* and Branched alkane *(d)* increased with increasing desiccation duration, whereas Docosane and Branched alkane *(e)* decreased over the duration of desiccation (S3 Table). Other CHCs did not show significant temporal changes with increasing desiccation duration, despite exhibiting significant differences in the univariate analyses (S3 Table).

**Table 2:** Results of multivariate analysis of variance showing the effect of desiccation duration on hydrocarbon profiles of male *D. melanogaster* exposed to varying desiccation duration. Follow-up univariate ANOVA and post hoc comparisons were performed to test which CHC peaks differed across desiccation duration treatments.

Effect	Df	Wilk’s λ	*F _(125, 152.5)_*	*P*
Desiccation durations	5	0.003463517	2.63901	< 0.001
Residuals	54			
**Univariate ANOVA**				
Peaks		*F _(5,54)_*		*P*
Docosene		0.7585490		0.584
**Docosane**		2.8343354		**0.024**
Branched alkane *(a)*		1.0634571		0.391
Tricosene *(a)*		1.8055344		0.127
**Tricosene *(b)***		3.2289232		**0.013**
Tricosene *(c)*		2.1427483		0.074
Tricosane		2.3688389		0.051
Tetracosene *(a)*		1.3134402		0.272
**Tetracosene *(b)***		3.0657078		**0.017**
Tetracosene *(c)*		1.7665565		0.135
Tetracosane		1.0564879		0.395
Branched alkane *(b)*		0.7196990		0.611
Pentacosene (a)		0.5711086		0.722
Pentacosene (b)		1.6825419		0.155
Pentacosene (c)		1.8455590		0.119
Pentacosane		0.8205485		0.540
**Branched alkane *(c)***		3.8047233		**0.005**
Hexacosane		1.3195059		0.270
**Branched alkane *(d)***		6.2847672		**< 0.001**
Heptacosane		0.2425834		0.942
**Branched alkane *(e)***		2.6005002		**0.035**
Branched alkane *(f)*		0.4320802		0.824
**Branched alkane *(g)***		6.1568840		**< 0.001**
Nonacosane		2.0070608		0.092
Branched alkane *(h)*		1.3001999		0.278

### Key CHC contributors of desiccation driven variation

The variable importance plot was used to identify the key CHCs aiding discrimination of the desiccation treatments. Among the CHC components, Branched alkane *(d),* Branched alkane *(c)* and Branched alkane *(g)* contributed substantially in predicting desiccation duration (Fig 2). Moderate importance was also observed for Docosane, Tetracosene *(b)* and Branched alkane *(e)*, while Tricosene *(b)* contributed relatively less (Fig 2).

**Fig 2:**
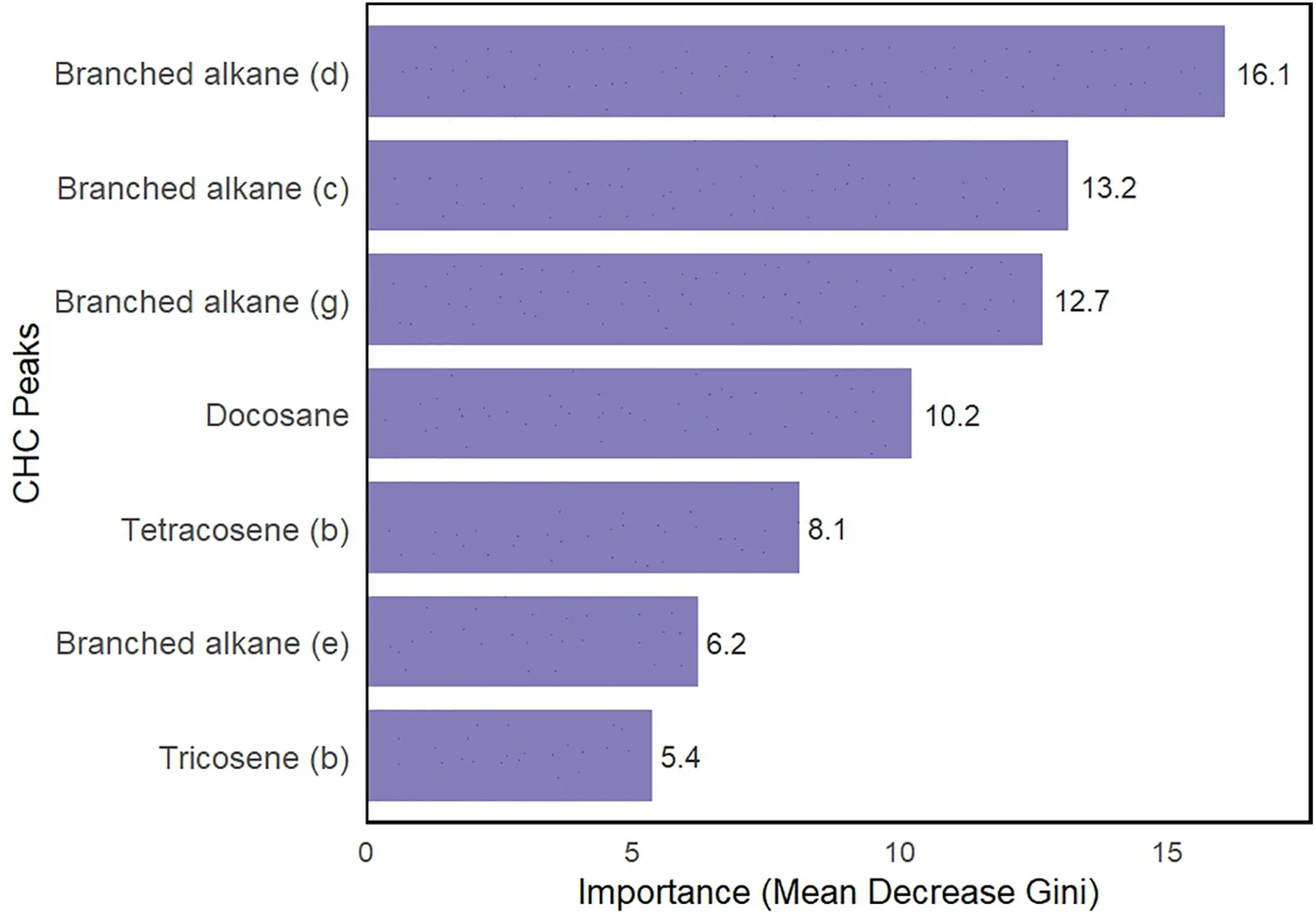
Variable importance plot showing the contribution of significant CHC components to multivariate differences across desiccation durations. Higher importance values indicate the contribution of CHC in predicting desiccation time-points in multivariate model (listed in decreasing importance Branched alkane *(d),* Branched alkane *(c)*, Branched alkane *(g),* Docosane, Tetracosene, Branched alkane *(e)* and Tricosene *(b).* The plot is based on CHC variables identified as significant in the MANOVA derived univariate tests.

### Temporal variation in cuticular hydrocarbon profiles

The variable importance plots (Fig 3, panels a-e) highlight CHCs that contributed most strongly to differentiate among desiccation durations. At 2 h (Fig 3, panel a), short-chain CHCs such as Tricosane and Tricosene *(b)* were dominant contributors in predicting desiccation duration. At 4 h, 6 h and 8 h (Fig 3 b, c, d) Branched alkane *(d)*, Branched alkane *(g)* and Docosane all contributed (refer to Fig 3 b, c, d). By 10 h (Fig 3 e), Branched alkane *(d)* showed a marked increase in importance, revealing a dynamic transition in CHC composition from shorter-chain to longer-chain hydrocarbons in response to prolonged desiccation.

**Fig 3:**
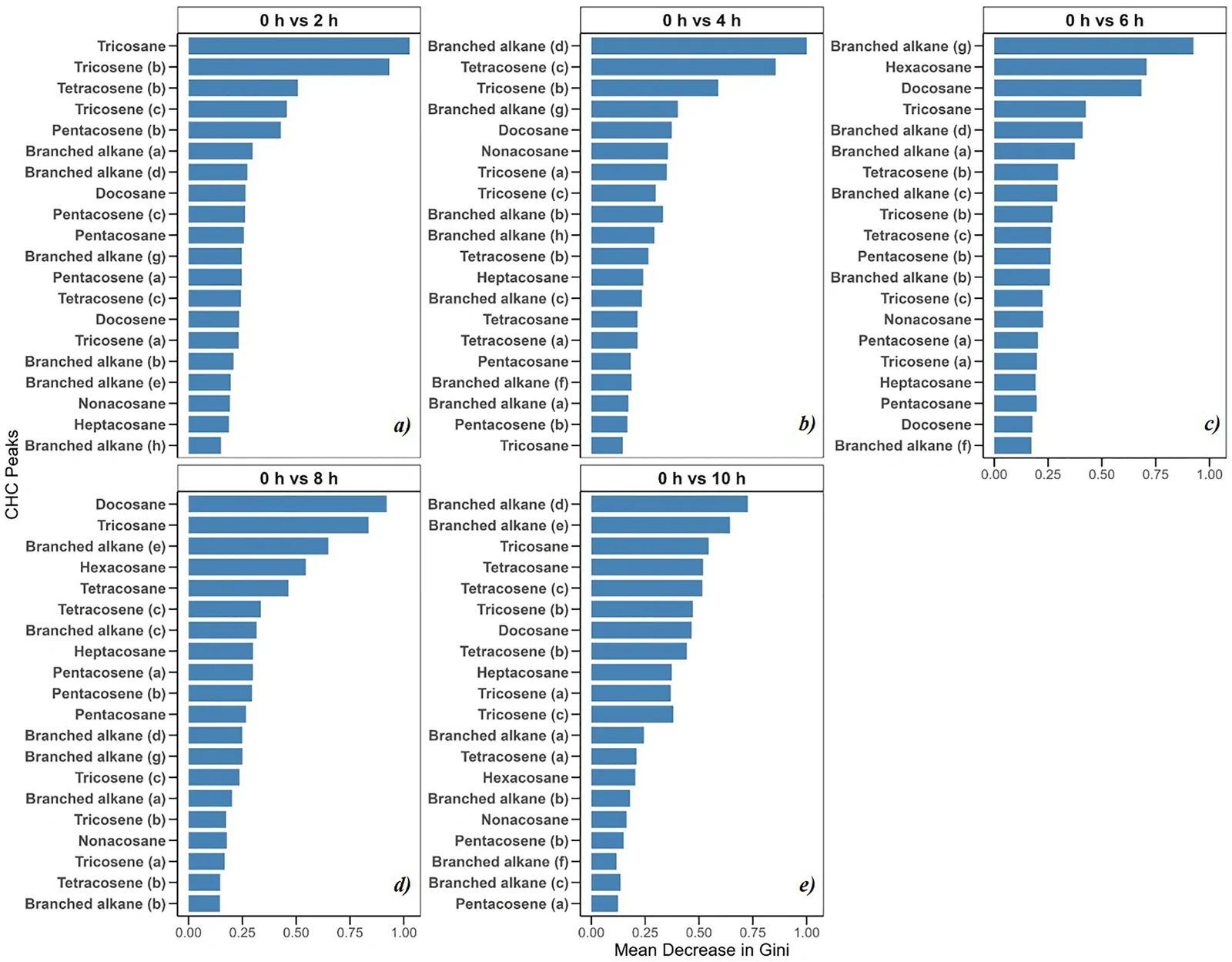
Cuticular hydrocarbon composition was used to identify the key contributors of desiccation duration. The variable importance from the random forest model was used to identify CHCs that varied most strongly between the control and desiccation treatments. Higher importance values indicate a greater contribution in predicting desiccation duration. The analysis was performed on untransformed CHC relative abundance values. The top 20 CHCs have been ranked from lower to higher importance and most of the CHCs have a small contribution to the accuracy of the model for desiccation duration. The X-axis represents the importance of each CHC compound in classification, and the Y-axis corresponds to the CHCs in order of increasing importance. Panels (a-e) show comparisons between control (0 h) and different desiccation time-points: 0, 2, 4, 6, 8 and 10 h, respectively.

### Effect of desiccation duration on cumulative water loss

One-way analysis of variance revealed a significant effect of desiccation durations on cumulative water loss (F_(5, 54)_ = 26.77; *P* < 0.001), with cumulative water loss increasing over time. Tukey’s HSD post hoc comparisons showed that all desiccation time-points differed significantly from 0 h (control): 2 h (estimate = −0.528, 95% CI: −0.757 to −0.299, *P* < 0.001), 4 h (estimate = −0.580, 95% CI: −0.809 to −0.351, *P* < 0.001), 6 h (estimate = −0.620, 95% CI: −0.849 to −0.391, *P* < 0.001), 8 h (estimate = −0.706, 95% CI: −0.935 to −0.477, *P* < 0.001) and 10 h (estimate = −0.815, 95% CI: −1.044 to −0.586, *P* < 0.001). Additionally, the 10 h group differed significantly from both 2 h (estimate = −0.287, 95% CI: −0.516 to −0.057, *P* = 0.0065) and 4 h (estimate = −0.235, 95% CI: −0.464 to −0.005, *P* = 0.042), indicating progressive increases in water loss with longer desiccation exposure. Overall, flies tended to lose more water with increasing desiccation duration (Fig 4).

**Fig 4:**
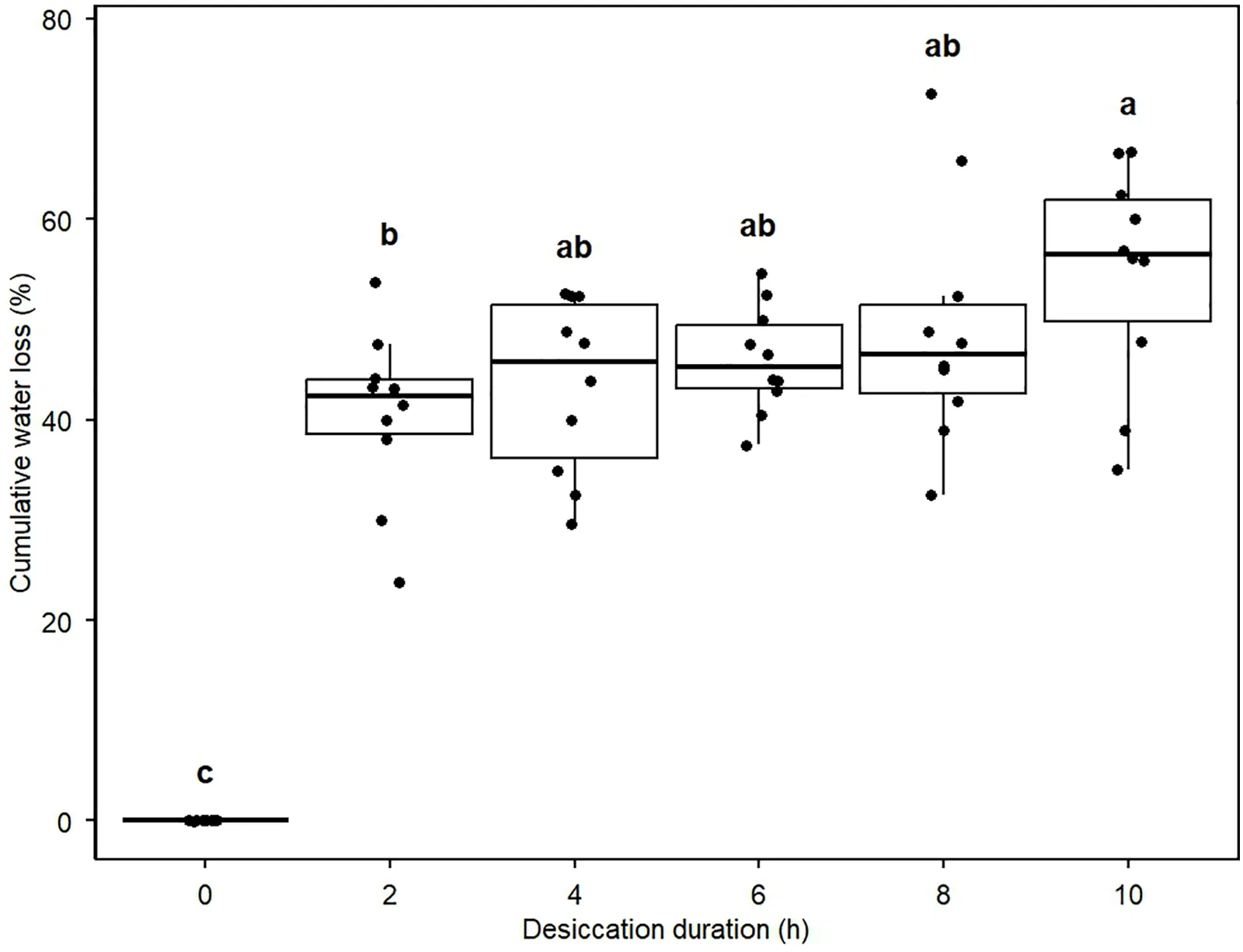
Cumulative water loss (%) in male *D.* melanogaster during desiccation progression. The box plot shows cumulative water loss (%) in male *D. melanogaster* (using Wharton’s method) during desiccation progression, with 0 h as the baseline. Different letters above the boxes denote statistically significant differences among desiccation time-points based on One-way ANOVA followed by Tukey’s HSD post-hoc comparisons (*P < 0.05).* The X-axis represents different desiccation durations (h) and the Y-axis represents cumulative water loss (%).

## Discussion

Our study reveals clear changes in the CHC profiles of male *D. melanogaster* in response to increasing durations of experimental desiccation. Consistent with our expectations, CHC profiles shifted rapidly under dry conditions, with distinct transitions observed as early as 2 h into drying, and these became more pronounced by 10 h of exposure to desiccating conditions. These temporal CHC shifts were mirrored by a progressive increase in cumulative water loss during desiccation, aligning with changes in CHC chain-length composition over time. Together, these patterns suggest a strong functional link between chemical plasticity in hydrocarbon composition and evaporative water loss, whereby desiccation exposure induces dynamic CHC composition shifts toward longer chain, less-volatile CHCs that enhance cuticular waterproofing during prolonged desiccation.

The role of CHCs in desiccation resistance have been reported in many insects [12,30,46]. These hydrophobic molecules are rapidly synthesised and deployed onto the outer integument, primarily to minimize cuticular transpiration and ensure water proofing [31,47]. The water-proofing ability of CHCs depends on their physicochemical properties [48,49], and previously, shifts in CHC profiles in response to desiccation stress and temperature have been demonstrated [46]. Unfortunately, how rapidly CHCs are synthesized as desiccation progresses remains less well understood.

Our findings revealed major changes in CHC profiles after 2 h and 4 h of experimental desiccation. In line with this pattern, cumulative water loss increased significantly across these treatment durations. Variable importance analysis comparing different desiccation durations identified short-chain CHCs, Tricosane and Tricosene *(b)* were the primary contributors in the early hours of desiccation (2 h), whereas longer methyl-branched CHCs, Branched alkane *(d)* and Branched alkane *(e)* were the primary contributors during later hours of desiccation (10 h). Consistent with this shift, linear trend analysis revealed that the abundance of long-chain methyl branched hydrocarbons, Branched alkane *(c)* and Branched alkane *(d)* increased with desiccation duration (S3 Table). These findings suggest a rapid temporal changes in CHC composition in response to desiccation progression. Similar associations were established between methyl-branched CHCs and desiccation tolerance in a previous large comparative physiology study involving 50 different *Drosophila* and related species, in which long methyl-branched CHCs were associated with desiccation tolerance [31]. Our findings are broadly consistent with this work.

The highest water loss occurred within the first 2 h of desiccation exposure. This is also comparable to water depletion patterns in male black field cricket (*Teleogryllus oceanicus*), where relatively greater water loss was observed during the early stages of desiccation [50]. Notably, water loss plateaued around 4, 6, and 8 h with no significant differences among these time-points suggesting a temporary stabilization in the water loss. This stabilization was likely because of the dominance of long chain hydrocarbons at 4 and 6 h (Fig 3), which enhance waterproofing and reduce water loss. These patterns indicate dynamic physiological responses occurring during prolonged desiccation. However, by 8 h of exposure, the hydrocarbon profiles shift towards short chain CHCs despite a plateau in cumulative water loss (Fig 4). This hydrocarbon turnover likely reflects continuous rapid re-organisation of CHC composition in response to prolonged desiccation. Notably, Stinziano, Sové (21) showed that CHC quantity and profile influence cuticular permeability, supporting that dynamic CHCs re-organisation directly contributes to reduction in evaporative water loss.

The observed dynamic shifts in CHC profiles seemingly reflect adaptive changes in HC composition under desiccating conditions. The CHC modifications documented here apparently increase waterproofing by altering cuticular properties associated with evaporative water loss. Our results suggest a phased response to desiccation; where an initial period of rapid water loss during early exposure was followed by a mid-phase (4–6 h) with a marked increase in long chain CHCs, conferring tighter regulation of water loss (Fig 3 and Fig 4). However, prolonged desiccation (8 h) was characterised by short chain CHCs, indicating physiological plasticity and constraints in hydrocarbon turnover [51] (Fig 3). However, such rapid plastic response could involve trade-offs with other life history traits (e.g., mating and survival) [52–54]. Given the role of CHCs in chemical communication, transitions in CHC profiles under desiccating conditions may affect mate recognition ability, resulting in altered mating success [37]. A recent study on male field cricket (*Teleogryllus commodus)* has shown that natural selection operating on CHCs via evaporative water loss can be strong, and favours increased total abundance and long-chain length CHCs, while sexual selection favours lower CHC abundance for maximised mating success [50]. Similar findings have been reported for *D. simulans* [54]. Interestingly, certain CHCs play a dual role in *Drosophila,* for instance, helping in water retention as well as mate choice [55,56]. Notably, two closely related rainforest *Drosophila* species, males experimentally perfumed with a methyl branched hydrocarbon (mbCHC; MeC_26_) exhibited both increased mating success and desiccation tolerance [56]. Such rapid changes in CHCs could also be helpful in exploring a wide range of habitats via processes such as acclimation [21,30]. *D. melanogaster* has a cosmopolitan status and a global distribution, success to such wide migration and colonization can possibly be attributed to dynamic properties of cuticle (i.e., CHCs).

While we observed rapid CHC changes during prolonged desiccation; the underlying mechanisms remain unclear. CHC biosynthesis is regulated by elongase and desaturase enzymes [57]. The activity of these enzymes might increase under desiccation stress and lead to directional synthesis of certain CHCs, which needs to be further evaluated. However, although our findings suggest rapid profile changes in CHCs under desiccation stress through time, we cannot conclusively state that these changes are a direct result of active CHC regulation. Previous studies have shown rapid changes in CHC profiles in response to social interactions (such as exposure to females) and environmental cues (light, temperature, and time of day) [58,59], as well as seasonal changes [52], indicating dynamic CHC regulation. Nevertheless, in this study, we did not directly test whether transition in CHC profiles was due to active CHC regulation or desiccation exposure. Further work involving gene expression profiling of CHC biosynthesis and oenocyte-specific enzyme activity is needed for clearer understanding of CHC regulation under water stress.

A limitation of our study is that we did not include parallel control groups maintained under non-desiccating, humid conditions and sampled at the same time points (2–10 h) as the desiccation treatments. Consequently, we cannot completely exclude the possibility that some of the temporal changes observed in CHC profiles were influenced by processes occurring independently of desiccation, including diel or circadian variation. CHC profiles in male *D. melanogaster* have been shown to exhibit reproducible variation over a single day, with turnover of some CHCs occurring within a few hours [58]. Age-related changes in CHC composition have also been documented in this species [60]. However, age is unlikely to be a major confounding factor in the present study because all flies were the same age at the start of the experiment and all treatments were conducted within a single day; moreover, the reported age-related changes occur over substantially longer timescales than the 10-h experimental period [60]. Future studies should therefore include time-matched control groups maintained under humid conditions and sampled concurrently with desiccated flies. Controlling the timing of treatment and sampling across the light–dark cycle would further help distinguish desiccation-specific changes in CHC composition from diel or circadian variation. Such an experimental design would provide a stronger test of whether the temporal shifts observed here are specifically induced by desiccation. Nevertheless, the temporal patterns observed in our study are consistent with previous work demonstrating rapid CHC responses to desiccation in *D. melanogaster* [31] (and see discussion above). Together with the parallel temporal changes in water loss, these findings support the interpretation that CHC plasticity may contribute to the response to dehydration exposure.

Finally, hydrocarbons are synthesized in oenocytes, specialised cells implicated in regulating lipid metabolism and production [61–65]. These cells contribute to epidermal waterproofing, thereby reducing cuticular transpiration and maintain fluid homeostasis [61]. The regulatory pathways governing CHC biosynthesis during desiccation across different developmental life stages could be crucial to our understanding of CHC deployment across the cuticle under stress.

## Conclusion

Our study demonstrated changes in CHC composition in male *D*. *melanogaster* in response to experimental desiccation. Cumulative water loss significantly increased across desiccation duration with substantial water loss evident during early exposure (2–4 h), which declined progressively as desiccation exposure progressed. Correspondingly, significant temporal changes in CHC profiles were observed, suggestive of dynamic re-organisation of epicuticular hydrocarbon layer during desiccation. Together, these findings are consistent with CHC plasticity in response to dehydration stress. Understanding how these findings apply to natural settings where insects experience fluctuating environmental conditions, is crucial to our understanding of insect responses to climate change.

## Supporting information

S1 Raw Data Raw data underlying the analyses presented in this study. The dataset contains relative abundances of 25 cuticular hydrocarbon (CHC) peaks from male *Drosophila melanogaster* across the experimental groups (0, 2, 4, 6, 8, and 10 h), together with data used for the analysis of body mass and water loss.
(XLSX)

S1 README README file accompanying the raw data. Description of the dataset structure, variables, experimental groups, and calculation of CHC relative abundance and water-loss measures.
(TXT)

S2 Table Tukey HSD post hoc comparisons for CHCs showing significant effects of desiccation duration in ANOVA. Pairwise comparisons among desiccation durations are presented with estimates, standard errors, *t*-values, and Tukey-adjusted *P*-values.
(DOCX)

S3 Table Linear regression-based temporal trend analysis of CHCs identified as significant in univariate ANOVA followed by Tukey HSD comparisons. For each CHC, the slope (β), standard error (SE), *t*-statistic, *P*-value, and direction of the temporal trend across desiccation durations are reported. Significant CHC peaks are highlighted in bold. The data set and accompanying code are available at Zenodo via https://doi.org/10.5281/zenodo.22079185.
(DOCX)
