# Supplementary material for: Temporal shifts in insect cuticular hydrocarbons in response to experimental desiccation
Source: PLoS One. 2026 Oct 13;21(10):e0359617. doi: 10.1371/journal.pone.0359617 (PMC13660911; doi:10.1371/journal.pone.0359617)
Supplement: Supplementary file 1 [file pone.0359617.s003.docx]

**S2 Table.** Tukey HSD post hoc Comparisons for CHCs showing significant effects of desiccation duration in ANOVA.

| **CHC Peak** | **CHC formula** | **Desiccation durations** | **Estimate** | **SE** | **t value** | **Adj. p-value (Tukey)** |
| --- | --- | --- | --- | --- | --- | --- |
| **Branched alkane *(c)*** | MeC_25_ | 2 -10 | -0.218 | 0.055 | -3.932 | 0.003 |
|  |  | 2 -6 | -0.180 | 0.055 | -3.249 | 0.023 |
|  |  | 2 - 4 | -0.176 | 0.055 | -3.173 | 0.028 |
|  |  |  |  |  |  |  |
| **Branched alkane *(d)*** | MeC_26_ | 2 - 10 | -0.195 | 0.040 | -4.903 | 0.000 |
|  |  | 0 - 10 | -0.182 | 0.040 | -4.578 | 0.000 |
|  |  |  |  |  |  |  |
| **Docosane** | C_22_ | 0 - 10 | 0.177 | 0.049 | 3.632 | 0.007 |
| **Branched alkane *(g)*** | MeC_28_ | 2 - 6 | 0.349 | 0.075 | 4.640 | 0.000 |
|  |  | 6 - 10 | -0.307 | 0.075 | -4.078 | 0.001 |
|  |  | 0 - 6 | 0.275 | 0.075 | 3.660 | 0.007 |
|  |  | 2 - 4 | 0.238 | 0.075 | 3.165 | 0.029 |
|  |  | 6 - 8 | -0.225 | 0.075 | -2.994 | 0.045 |
|  |  |  |  |  |  |  |
| **Tricosene *(b)*** | C_23:1 (b)_ | 0 - 2 | 0.157 | 0.047 | 3.332 | 0.018 |
|  |  | 0 - 10 | 0.143 | 0.047 | 3.030 | 0.041 |
| **Tetracosene *(b)*** | C_24:1 (b)_ | 6 - 10 | 0.233 | 0.067 | 3.503 | 0.011 |
