# Supplementary material for: Temporal shifts in insect cuticular hydrocarbons in response to experimental desiccation
Source: PLoS One. 2026 Oct 13;21(10):e0359617. doi: 10.1371/journal.pone.0359617 (PMC13660911; doi:10.1371/journal.pone.0359617)
Supplement: Supplementary file 2 [file pone.0359617.s004.docx]

**S3 Table**. Linear regression based temporal trend analysis of CHCs that are identified as significant in Univariate ANOVA followed by Tukey HSD comparisons. For each CHC, the slope (β), standard error (SE), t-statistic, and *P-value* indicating changes in CHCs across desiccation durations. Significant CHC peaks are highlighted bold.

| **CHC peak** | **Slope (β)** | **SE** | **t** | **P** | **Temporal trend** |
| --- | --- | --- | --- | --- | --- |
| **Branched alkane *(d)*** | 0.016534297 | 0.003450498 | 4.7918589 | **0.0000119** | Increase |
| **Docosane** | -0.013860747 | 0.004076846 | -3.3998697 | **0.0012300** | Decrease |
| **Branched alkane *(e)*** | -0.012737184 | 0.003764876 | -3.3831618 | **0.0012900** | Decrease |
| **Branched alkane *(c)*** | 0.012883757 | 0.004979568 | 2.5873245 | **0.0122000** | Increase |
| Tetracosene *(b)* | -0.009989503 | 0.006008814 | -1.6624749 | 0.1020000 | Decrease |
| Tricosene *(b)* | -0.005920395 | 0.004318106 | -1.3710630 | 0.1760000 | Decrease |
| Branched alkane *(g)* | -0.004641302 | 0.007659087 | -0.6059862 | 0.5470000 | Decrease |
